# Hemothorax resulting from an initially masked aortic perforation caused by penetration of the sharp edge of a fractured rib: A case report

**DOI:** 10.1016/j.ijscr.2020.02.023

**Published:** 2020-02-13

**Authors:** Yasufumi Goda, Tsuyoshi Shoji, Naoki Date, Hiromichi Katakura

**Affiliations:** Department of Thoracic Surgery, Otsu Red Cross Hospital, Shiga, Japan

**Keywords:** CT, computed tomography, POD, postoperative day, BTAI, blunt traumatic aortic injury, TEVAR, thoracic endovascular aortic repair, Blunt chest trauma, Hemothorax, Aortic injury, Rib fracture

## Abstract

**Introduction:**

There are multiple causes of hemothorax in blunt chest trauma. However, a traumatic hemothorax with an uncertain cause is potentially life-threatening without treatment, because an undetected and hidden great vessel injury can remain unknown. Delayed diagnosis can lead to death.

**Presentation of case:**

A 77-year-old man was transferred to a local hospital, after experiencing a 3-m fall. Contrast CT of the chest revealed a left clavicle fracture, multiple left rib fractures and hemopneumothorax, but no obvious signs of great vessel injury, such as aortic injury. His condition was stable, owing to the chest tube thoracostomy with 800 ml blood output and intravenous fluid. The patient was then transferred to our hospital for further treatment. However, his condition rapidly deteriorated in the ambulance on the way to our hospital, and he needed a blood transfusion. On arrival, he was in shock, with his vital signs compromised due to blood loss. Emergency open thoracotomy was performed to explore the bleeding point and stop hemorrhaging. Intraoperative findings revealed sharp edges of the fractured fourth and fifth left ribs to be protruding into the chest cavity toward the descending aorta and causing an aortic pinhole injury. Ruptured aorta was repaired with a pledget-armed sutures and the sharp fractured ribs were resected. The patient was discharged, uneventfully, 35 days after the operation.

**Conclusion:**

This case suggests that even if great vessel injury is not detected on contrast CT at admission, it should always be considered especially in a hemothorax case with multiple rib fractures.

## Highlights

• Rare aortic injury from a rib fracture, which is not found in primary evaluation.
• Hemothorax with great vessel injury can be fatal and should be detected using computed tomography.
• Some great vessel injuries cannot be detected through contrast-enhanced chest computed tomography.
• Great vessel injury should be considered in hemothorax with multiple rib fractures.

## 1 Introduction

Initial management of hemothorax in blunt chest trauma includes identification and treatment of life-threatening injuries, control of bleeding, and resuscitation. Despite several causes of hemothorax following blunt chest trauma, great vessel injury can be fatal and should be detected at the earliest possible time in order to save patients’ lives. In patients with blunt traumatic aortic injury (BTAI), chest contrasted computed tomography (CT) is the gold standard diagnostic imaging modality [1,2]. In fact, recent evidence-based guidelines recommended CT over aortography for the diagnosis of BTAI [2,3]. However, achieving accurate diagnosis without signs of extravasation, dissection, laceration or pseudoaneurysm in contrasted CT is difficult. Here, we report a rare case of aortic injury caused by the penetration by the sharp edge of a fractured rib, which went undetected on CT of primary evaluation.

This work has been reported in line with the SCARE criteria [4].

## 2 Presentation of case

**Fig. 1:**
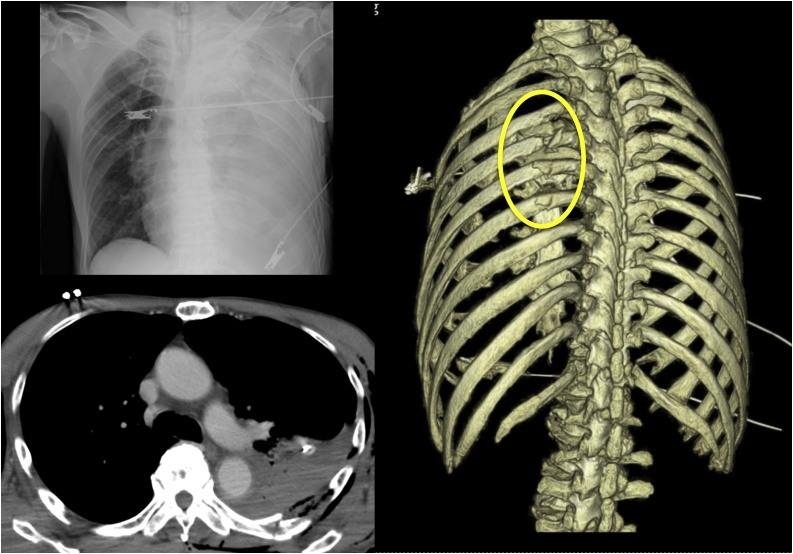
Chest radiography and computed tomography (CT) performed in a supine position on admission in the local hospital. Chest radiography and CT showed multiple left rib fractures and massive hemothorax. The fractured fourth to seventh ribs are shown on three-dimensional (3D) chest CT scan.

**Fig. 2:**
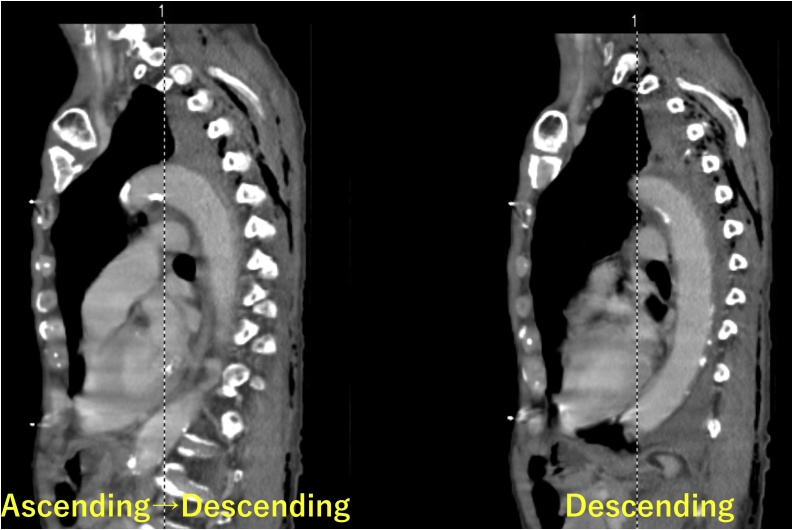
Late equilibrium phase contrasted computed tomography (CT) on admission in the local hospital. Sagittal CT cut showing no sign of aortic injury, such as extravasation, dissection, or pseudoaneurysm.

After being injured due to a 3-m fall, a 77-year-old man was initially taken to the local hospital, which is an hour’s drive from our hospital. In the primary survey, the patient was found to be hemodynamically stable and responded to primary care comprising of rapid intravenous fluid therapy. Radiography performed in the supine position showed complete left lung field opacification. Chest CT revealed multiple left rib fractures, left clavicle fracture, and hemopneumothorax (Fig. 1), with no signs of great vessel injury, such as extravasation, dissection, or pseudoaneurysm (Fig. 2). The hemoglobin value of the patient was 10.6 g/dl, moderately down 2.8 points from his usual value. The patient was stabilized by draining 800 ml of blood from a chest drain and fluid therapy. After the stabilization of the patient during the primary survey, the doctors in the local hospital decided to transfer him to our hospital for further management, including surgical intervention because they assumed that hemothorax could be caused by intercostal arterial injuries, lung contusion or great vessel injuries from multiple fractured ribs, as indicated by the chest X-ray radiographic appearance, showing a total opacification and the high initial drain output.

On the way to our hospital, the patient’s condition deteriorated in the ambulance regardless of the stabilization measures taken during the primary survey. His condition was unstable, although his blood pressure responded to intravenous fluids and blood transfusion in the ambulance.

On arriving at our hospital, the patient was confused and in shock, with a pulse rate of 121 beats per minute, blood pressure of 92/46 mmHg, and 100% oxygen saturation with oxygen therapy (10 L/min, reservoir mask). There was a total blood output of 2500 ml from the chest tube 3 h after the injury. Regardless of blood transfusion, the patient was in persistent shock and his vital signs were compromised due to blood loss. His condition deteriorated so rapidly that we were unable to perform a reevaluation with contrast chest CT to identify the bleeding site.

**Fig. 3:**
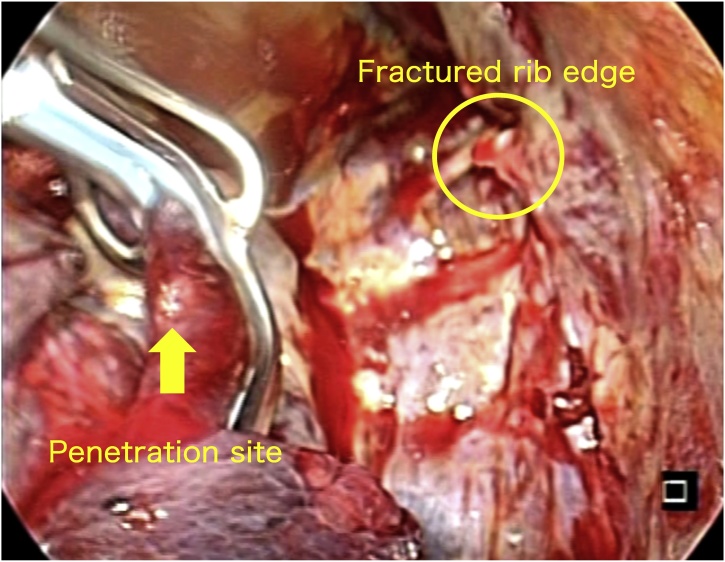
Intraoperative image. The yellow arrow shows the location of the 4-mm puncture wound in the descending aorta adjacent to the edge of the fourth and fifth broken ribs. The aorta was side-clamped and repaired with a pledgeted suture.

Because of his hemodynamic instability and respiratory insufficiency, with continuous bleeding from the chest tube, we performed emergency open thoracotomy to identify the bleeding point and stop the bleeding. On performing left thoracotomy, massive hemorrhage and clots with approximately 2,000 ml of fresh blood were found in the thorax. We quickly explored the site for the bleeding point, but occurrence of a cardiac arrest necessitated manual cardiac massage and bolus injection of fluid and catecholamine. We then identified the bleeding point in the descending aorta with blood outflow synchronized with cardiac massage. The patient eventually stabilized after digital compression of the bleeding point and intensive management followed by blood transfusion. Intraoperative findings revealed protruding sharp edges of the fractured fourth and fifth left ribs into the chest cavity toward the descending aorta, with the aortic pinhole injury, probably resulting from a momentary penetration of the sharp edge of the fractured rib (Fig. 3).

The aorta was side-clamped and then repaired with a 5-0 Prolene™ (Ethicon, Somerville, NJ, USA) suture with pledget, and the sharp fractured ribs were trimmed.

Postoperatively, the patient was transferred to the intensive care unit for prolonged mechanical ventilation. On postoperative day (POD) 3, he was extubated successfully. Neurological evaluation was performed because of his cardiac arrest during the operation, and he subsequently recovered without complications. After the aortic reevaluation, the patient was discharged on POD 35 with no deficits.

Two years after the operation, the patient is known to be doing well.

## 3 Discussion

BTAI is the second most common cause of death in blunt trauma cases. Early diagnosis of BTAI is critical as 80% of patients would die before arriving at the hospital [2].

Although contrast CT is strongly recommended to diagnose BTAI [2,3], in this case, we were unable to detect an aortic injury using contrasted CT as the primary diagnostic method, regardless of the chest X-ray radiographic appearance, which showed a total opacification and the high initial drain output which are findings suggesting great vessel injuries. This was considered to be due to the rare pattern of the aortic injury, caused by a momentary penetration by the sharp edge of the fractured rib.

We assume the scenario of injury in this case to be as follows: the pointed edge of the fractured rib stuck sharply into the aorta and came out in a single moment. Actual diameter of the aortic injury site was found to be 4 mm by intraoperative findings, which cannot be detected on CT. Furthermore, the aortic puncture site was acting like a flap, and was covered with blood clots, which stopped the bleeding and masked the signs of aortic injury during contrast extravasation. While transferring the patient to our hospital, the clots covering the aortic pinhole injury could have been peeled off, resulting in rapid worsening of his condition.

In an unstable situation, regardless of the stabilization during the primary survey, transferring the 77-year-old hemothorax patient 1 h’s drive away was risky. However, this was associated with local organizational issues. The local hospital does not have a cardiothoracic surgery department.

Emergency staff in the local hospital performed precise primary survey for the patient. The radiographic findings from the contrasted CT, indicated an uncertain cause for the hemothorax, but they assumed that the hemothorax could have been caused by intercostal vessel injuries, lung contusion, or great vessel injuries, requiring further investigations and management, including surgical intervention. Ideally, further investigations and management should have been performed in the local hospital; however, our hospital is the only hospital in our district equipped with an emergency and trauma care center with a cardiothoracic surgical department.

The emergency medical staff in local hospital rode on the ambulance together with the patient while transferring the patient to our hospital. They dealt with the critical situation, by performing the required blood transfusion, without this care the patient would be dead.

If we had been able to detect his aortic injury preoperatively, thoracic endovascular aortic repair (TEVAR) would have been the first choice of treatment, as it does not require open thoracotomy and double-lumen endotracheal tube insertion for separate lung ventilation or a right decubitus position to prevent a dreadful rupture in the operation room. In fact, TEVAR has been recommended for BTAI without contraindication over open thoracotomy, according to the current guidelines because of the lower mortality rates and favorable outcome [2,5,6].

Retrospectively, re-evaluation of contrasted CT with early arterial phase or an aortogram could have been obtained preoperatively, but the patient’s condition was deteriorating so rapidly that we did not have enough time to wait for further evaluation to detect the aortic injury. In a situation similar to this case, Tsai et al. previously reported that a more aggressive management strategy with surgical exploration after CT, rather than waiting for an aortogram, might have enabled their patient to survive [7].

Regarding the etiology of this case, there have been several reports of aortic injury caused by the sharp edges of fractured ribs, and in all cases, the injured sites of ribs were left-sided posterior rib fractures [8,9]. The location of left-sided posterior rib fractures might be a risk factor for this type of aortic injury, considering the anatomical location in the thorax.

## 4 Conclusion

In hemothorax patients, direct aortic penetration injury caused by a fractured rib segment is rare. This case suggests that even when great vessel injury, including aortic injury, goes undetected at admission, it should always be considered as a possibility in patients with hemothorax, especially those with multiple rib fractures.

## Funding

We have no disclosures or financial support for this study.

## Ethical approval

This case report is not research study, therefore approval was not given. Ethical approval has been exempted by our institution, Otsu Red Cross Hospital.

## Consent

Written informed consent was obtained from the patient for publication of this case report and any accompanying images.

## Author contribution

Yasufumi Goda: study concept, data collection, interpretation, writing the paper.

Tsuyoshi Shoji: study concept, review of the paper.

Naoki Date: date collection.

Hiromichi Katakura: study concept, review of the paper.

All authors participated in the patient’s care, performed surgeries, and read and approved the final manuscript.

## Registration of research studies

Since this is a case report, not a research study, registration is not indicated.

## Guarantor

All authors are guarantors for this study.

## Provenance and peer review

Not commissioned, externally peer-reviewed.

## Declaration of Competing Interest

The authors declare that they have no competing interests.

## References

[1] Trust M.D., Teixeira P.G.R. (2017). Blunt trauma of the Aorta, Current Guidelines. Cardiol. Clin..

[2] Fox N., Schwartz D., Salazar J.H., Haut E.R., Dahm P., Black J.H. (2015). Evaluation and management of blunt traumatic aortic injury: a practice management guideline from the Eastern Association for the Surgery of Trauma. J. Trauma Acute Care Surg..

[3] Fabian T.C., Davis K.A., Gavant M.L., Croce M.A., Melton S.M., Patton J.H. (1998). Prospective study of blunt aortic injury: helical CT is diagnostic and antihypertensive therapy reduces rupture. Ann. Surg..

[4] Agha R.A., Borrelli M.R., Farwana R., Koshy K., Fowler A., Orgill D.P., For the SCARE Group (2018). The SCARE 2018 statement: updating consensus surgical CAse REport (SCARE) guidelines. Int. J. Surg..

[5] Elkbuli A., Dowd B., Spano I.I.P.J., Smith Z., Flores R., McKenney M. (2019). Thoracic endovascular aortic repair versus open repair: analysis of the National Trauma Data Bank. J. Surg. Res..

[6] González S.S., Martín A.C., Moñux G.D., Martínez I.L., Hernando M.R., Serrano F.H. (2013). Stent-graft repair for blunt traumatic aortic injury: functional and survival outcomes. Int. Angiol..

[7] Tsai F.C., Chang Y.S., Lin P.J., Chang C.H. (1999). Blunt trauma with flail chest and penetrating aortic injury. Eur. J. Cardiothoracic Surg..

[8] Bruno V.D., Batchelor T.J. (2009). Late aortic injury: a rare complication of a posterior rib fracture. Ann. Thorac. Surg..

[9] Boyles A.D., Taylor B.C., Ferrel J.R. (2013). Posterior rib fractures as a cause of delayed aortic injury: a case series and literature review. Injury Extra.

